# T1 mapping in discrimination between hypertrophic and hypertensive cardiomyopathy

**DOI:** 10.1186/1532-429X-16-S1-O61

**Published:** 2014-01-16

**Authors:** Rocio Hinojar, Benjamin P Goodman, Adriana Villa, Eduardo Arroyo Ucar, Darius Dabir, Tobias Schaeffter, Eike Nagel, Valentina Puntmann

**Affiliations:** 1 Cardiovascular Imaging Department, King's College London, London, UK, London, UK

## Background

The differential diagnosis of hypertrophic phenotype remains challenging in clinical practice, in particular between hypertrophy cardiomyopathy (HCM) and increased left ventricular wall thickness (LVWT) due to systemic hypertension. Diffuse myocardial fibrosis is the characteristic feature in HCM, whereas hypertensive response is underpinned by addition of myofibrils in otherwise normal myocardial tissue. Late gadolinium enhancement (LGE) imaging provided important new way of differentiation between these two entities by separating those cases with evidence of regional fibrosis. Whereas approximately 60% of patients with HCM reveal visually discernable LGE, T1 mapping is highly discriminative, irrespective of the presence of LGE.

## Methods

Sixty patients with diagnosis of unequivocally hypertrophic cardiomyopathy and fifty patients with hypertensive cardiomyopathy underwent routine cardiac MRI protocol including assessment of function and scar (3-Tesla). T1 values were measured conservatively within septal myocardium in midventricular short-axis slice prior to administration of 0.2 mmol/kg of gadobutrol.

## Results

**Figure 1:**
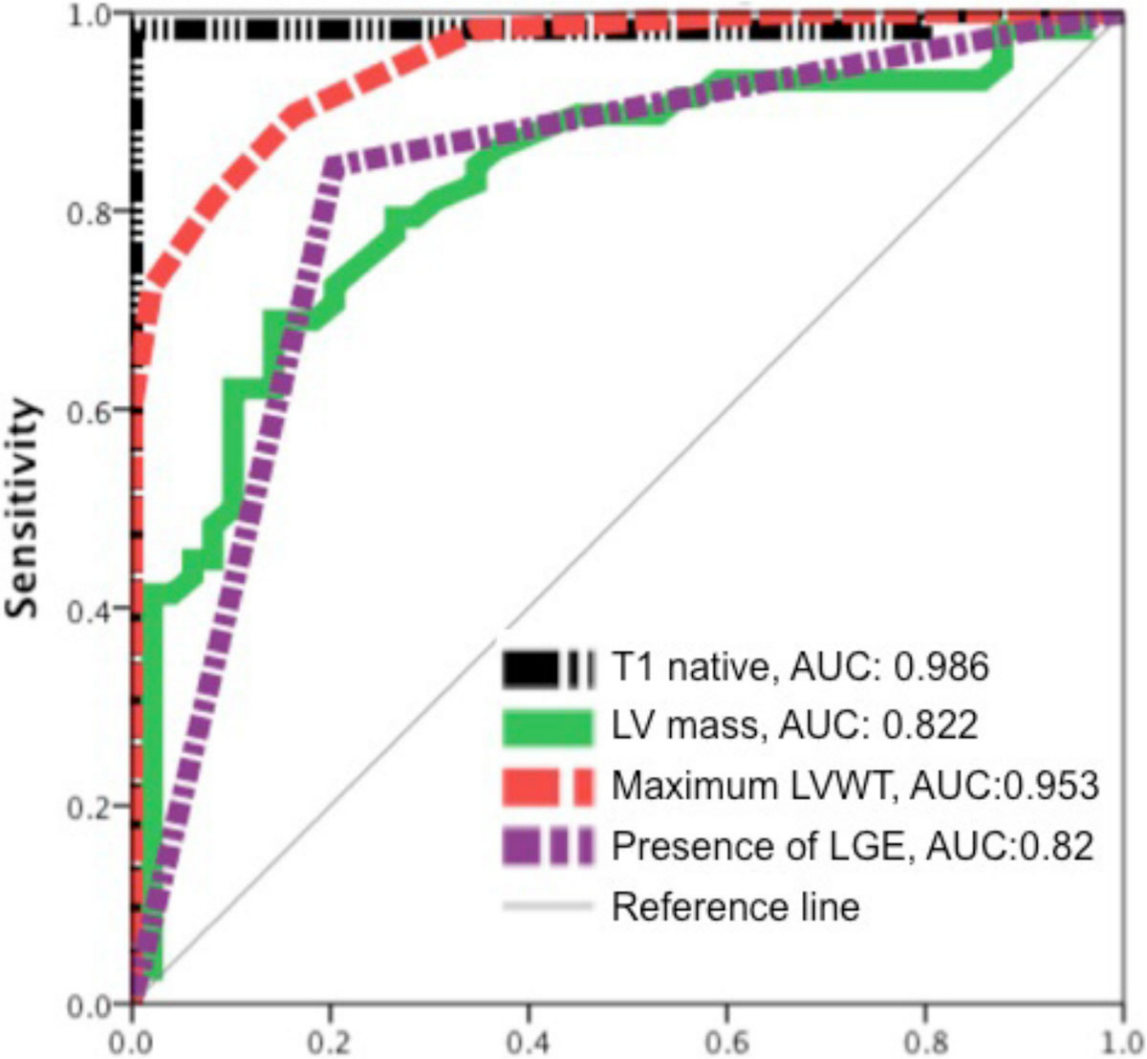
**Diagnostic Performance of T1 Mapping compared with LV mass, maximum LVWT and the presence of LGE in the differentiation between HCM and hypertensive cardiomyopathy**.

HCM group showed higher LV mass and maximum LVWT than the hypertensive group (HCM vs. hypertensive: LVmass, g/m2: 97 ± 31 vs. 69 ± 22; maximum LVWT 18.4 ± 3 vs. 13.3 ± 1.4, p < 0.0001). There was LGE in 20% of hypertensive (n = 10, 4 of them with an ischaemic pattern) and in 84% of hypertrophic cardiomyopathy (n = 50, 3 of them with an ischaemic pattern) (p < 0.001). Patients with HCM showed significantly higher T1 values compared to hypertensive patients (HCM vs. hypertensive, msec: 1164 ± 45 vs. 1049 ± 31, < 0.0001). Native T1 values were concordant to LVWT and LV mass (r = 0.52 and r = 0.46, p < 0.001, respectively). T1native held superior diagnostic accuracy compared to conventional functional parameters and the presence of LGE to discriminate between hypertrophic or hypertensive cardiomyopathy (Figure 1). T1 native was identified as an independent discriminator between the two conditions.

## Conclusions

We demonstrate that native T1 values can reliably discriminate between hypertrophic and hypertensive cardiomyopathy. We propose that native T1 may serve as a novel diagnostic marker to discriminate between the two hypertrophic conditions.

## Funding

We would like to acknowledge Department of Health via the National Institute for Health Research (NIHR) comprehensive Biomedical Research Centre award to Guy's & St Thomas' NHS Foundation Trust in partnership with King's College London and King's College Hospital National Health Service Foundation Trust. Dr. Rocio Hinojar was supported by the Fundacion Alfonso Martin Escudero.

